# CD59 receptor targeted delivery of miRNA-1284 and cisplatin-loaded liposomes for effective therapeutic efficacy against cervical cancer cells

**DOI:** 10.1186/s13568-020-00990-z

**Published:** 2020-03-17

**Authors:** Li Wang, Ting-Ting Liang

**Affiliations:** 1 Department of Pharmacy, Jining No. 1, People’s Hospital, Jining, 272011 Shandong China; 2 Department of Obstetrics and Gynecology, Weifang No. 2 People’s Hospital, No. 7 Yuanxiao Street, Kuiwen District, Weifang, 261041 Shandong China

**Keywords:** Cervical cancers, Liposomes, miRNA-1284, Cisplatin, Apoptosis

## Abstract

Co-delivery of two different therapeutics (miRNA-1284 and cisplatin (CDDP)) into the cancer cells in a single nanocarrier provides new dimension to the cancer treatment. In this study, we have designed the CD59sp-conjugated miRNA-1284/cisplatin(CDDP)-loaded liposomes for the enhanced therapeutic effect against cervical cancers. Compared with miRNA-1284/CDDP-loaded liposomes (LP-miCDDP), CD59 antibody-conjugated LP-miCDDP (CD/LP-miCDDP) showed a significantly higher cytotoxicity in HeLa cells. Notably, MiR-1284 showed a typical concentration-dependent cell killing effect in the cervical cancer cells owing to the downregulation of HMGB1. Flow cytometer analysis showed that CD/LP-miCDDP resulted in maximum apoptosis effect (~ 60%) compared to CDDP (~ 20%) or miR-1284 (~ 12%) treated cells indicating the superior anticancer effect in the cancer cells. Importantly, CD/LP-miCDDP significantly prolonged the blood circulation of encapsulated drug in rats with AUC_(o-t)_ of CD/LP-miCDDP showed a 6.9 fold higher value than that of free CDDP. Similarly, CD/LP-miCDDP showed an eightfold decrease in the clearance (CL) and 3.6-fold higher t_1/2_ compared to that of free CDDP. Overall, results demonstrated that targeted and synergistic co-delivery of therapeutic components could be promising in cervical cancer therapy.

## Introduction

Cervical cancer is one of the major world problems and considered to be one of the common gynecologic cancers making it as fourth most cancer related death among women (Kanavos 2006). Approximately, 85% of cervical cancer cases originate from developing countries such as China. According to one statistics, 87,982 people were diagnosed with cervical cancer in 2011 in China and more than 20,000 patients have been died in the same year (Jin et al. 2016). The global incidence of cervical cancer kept increasing with more than 250,000 deaths each year. Based on the guidelines of NCCN for cervical cancer, cisplatin (CDDP) is considered as a first line of treatment option (Siegel et al. 2014). CDDP alone or in combination with paclitaxel has been recommended for advanced or metastatic cancers. At times, treatment includes radiotherapy or surgery; however it has severe complications such as non-specificity and aggressiveness (Hirte et al. 2015). Besides, chemotherapy with CDDP or combination with other chemotherapeutic agents results in severe dose-limiting toxicity such as nephrotoxicity or hepatotoxicity and leads to severe drug resistance (Scatchard et al. 2012). Therefore, a potential therapeutic approach by which drug toxicity could be reduced and chemosensitivity of CDDP towards cervical cancer could be increased is perused continuously.

MicroRNAs (miR) are typically consists of a chain of 17–25 nucleotides, non-coding short RNAs and binds to the specific mRNAs at the 3′UTRs (Wang et al. 2015). Several of the miRNAs are indicated as key regulators in the proliferation, differentiation and regulation of cancer cell growth by acting on the gene expression at post-transcriptional level (Shin and Chu 2014). Studies have demonstrated that miRNAs can exhibit the control over cancer cells by controlling the key pathways (Ramasamy et al. 2019). In this regard, miR-1284 has been shown to inhibit the cancer cell growth by targeting the downstream targets (Li et al. 2017). Importantly, its expression has been related with the chemosensitivity in gastric cancers. Therefore, in this study, we have selected miR-1284 to increase the chemosensitivity of CDDP for the treatment of cervical cancers (Huang et al. 2017; Pan et al. 2016).

The free CDDP is known to exhibit severe toxicity to the vital organs due to non-specific distributions and thereby it has limited efficacy in the tumors. Similarly, miRNA is highly unstable in the systemic circulation. Keeping this in mind, nanocarrier-based drug delivery system received great attention owing the potential to improve the physicochemical characteristics of the anticancer drug (Oberoi et al. 2013; Panyam and Labhasetwar 2003). The nanocarriers are demonstrated to enhance the therapeutic efficacy, reduce the unwanted adverse effects, improve the blood circulation, and improve the cellular accumulation in the tumor tissues (Lockhart et al. 2015; Ramasamy et al. 2017). To be specific, liposome has emerged as one of the most tested carrier system in clinical trials (Ichihara et al. 2019). The unique structure of liposome provides high drug loading, surface modifications, plasma stability and tunable particle size. Although, liposome accumulates in the tumor tissue via enhanced permeation and retention (EPR) effect, targeted carrier remarkably increase the effectiveness of the therapy (Zhang et al. 2019). Tumor targeted carrier system selectively accumulate in the tumor and kill the cancer cells without causing unwanted side effects. Several receptors are expressed in the cervical cancers such as EGFR, CD44 or HER2 which could be utilized to target the nanoparticles (Day et al. 2017; Gasparetto et al. 2017). Recently, CD59 has been reported to overexpress in the cervical cancers and in other cancers, while is low expressed in normal cells (Afshar-Kharghan 2017). CD59 is a membrane regulatory component and promote immune escape of tumor cells (Li et al. 2017).

**Fig. 1:**
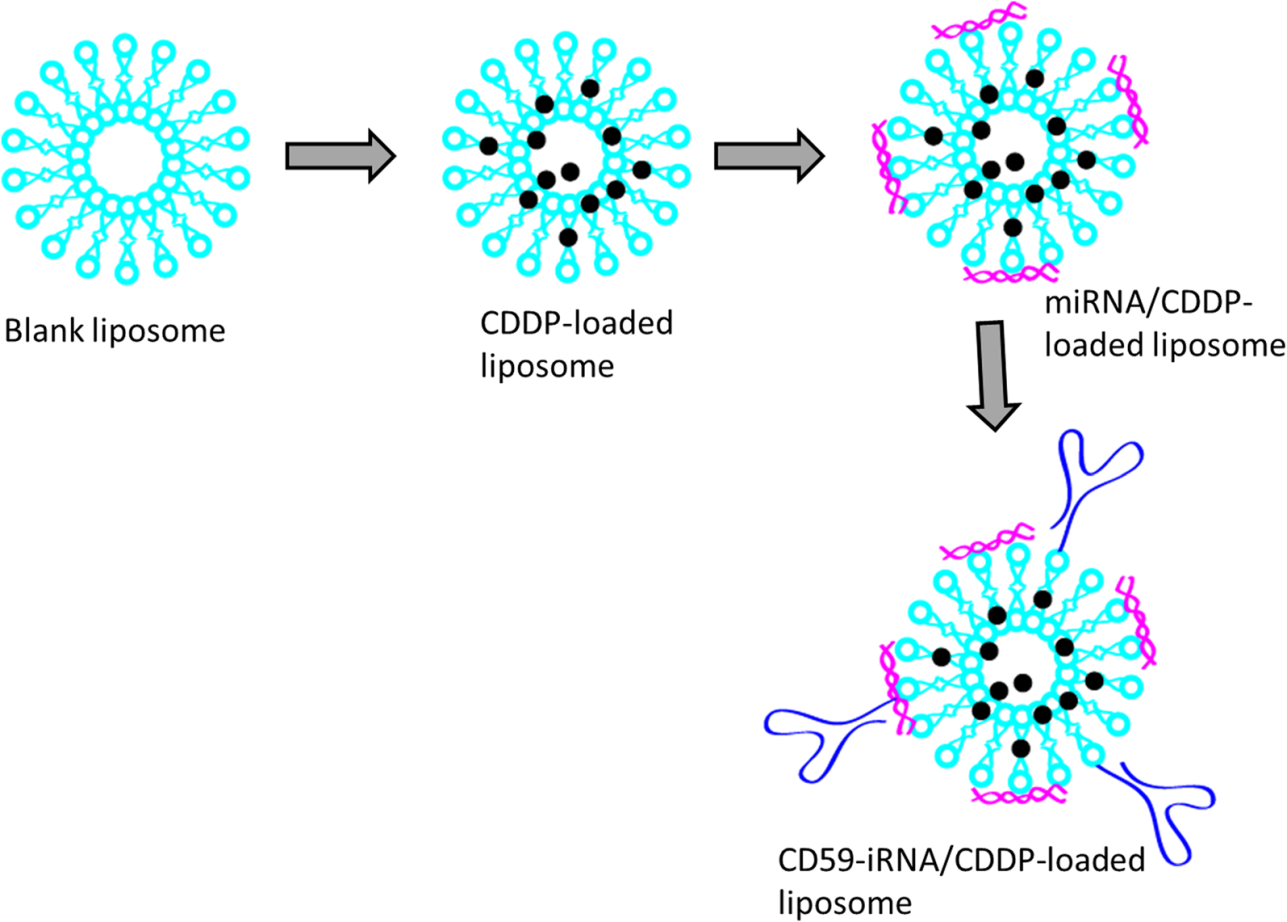
Graphical presentation of preparation of CD59sp-conjugated CDDP/miRNA-1284 liposome. The CDDP was loaded in the liposome shell and miRNA was loaded on the surface of liposomes

Therefore, we have designed unique CD59sp-conjugated CDDP/miR-1284-loaded liposomes for the effective treatment of cervical cancers (Fig. 1). The targeting specificity of CD59sp was evaluated by flow cytometry and confocal laser scanning microscopy (CLSM). The in vitro anticancer effect was evaluated by means of cell viability analysis, and apoptosis assay. Finally, pharmacokinetic analysis was performed to determine the blood circulation property of the designed carrier system.

## Materials and methods

Cisplatin (DDP) was purchased from Shandong Boyuan Pharmaceutical Co., Ltd (Ji’nan, China). Distearoylphosphatidylcholine (DSPC), distearoyl-N-(monomethoxy poly(ethylene glycol)succinyl)phosphatidylethanolamine (DSPE-PEG), distearoyl-N-(3-carboxy-propionoylpoly (ethyleneglycol) succinl) phosphatidylethanolamine (DSPE-PEG-COOH), and 1,2-dioleoyl-3-trimethyl-ammoniumpropane (DOTAP) were purchased from Avanti Polar Lipids, China. MiR-1284 was purchased from GenePharma Co., Ltd. (Shanghai, China). Dimehyl-2-thiazolyl)-2,5-diphenyl-2H-tetrazolium bromide (MTT). All other chemicals were of analytical grade.

### Preparation of CD59sp-conjugated CDDP/miRNA-loaded liposomes

At first, CDDP-loaded liposome was prepared by hydration-sonication method. Briefly, distearoylphosphatidylcholine (DSPC) succinylphosphatidylethanolamine (DSPE-mPEG), distearoyl-N-(3-carboxy-propionoylpoly (ethyleneglycol) succinl) phosphatidylethanolamine (DSPE-PEG-COOH), and 1,2-dioleoyl-3-trimethyl-ammoniumpropane (DOTAP) were added in a molar ratio of 40:20:10:2 in the chloroform (along with 10% w/w of CDDP). The organic solvent was evaporated in a rotary evaporator at 55 °C for 2 h. The dried lipid-film was hydrated by incubating in a 1X PBS for 1 h. The crude dispersion was sonicated for 5 min using a probe sonicator. To this liposome, 2 mg EDC was added per 1 mg/ml of liposome and stirred for 1 h; followed by CD59sp (1 mg/ml) was added to the solution and stirred in dark condition for 4 h. The unconjugated initial ingredients and unconjugated CD59 were removed by centrifugation at 5000×*g* for 15 min at 4 °C. The cationic liposome was then incubated with required amount of miRNA overnight and it is loaded on the surface of liposome.

### Characterization of CD59sp-conjugated CDDP/miRNA-loaded liposomes

The particle size and polydispersity index (PDI) and zeta potential was determined by dynamic light scattering (DLS) technique using ZetaSizer (Nano series- Nano-ZS ZEN3600, Malvern Instruments, UK). Before the measurement, 0.1 ml of samples was diluted to 1 ml and measured triplicate at 25 °C. The particle morphology was evaluated using transmission electron microscopy (TEM, JEM-2100, JEOL, Japan). The drop of samples were placed in the copper grid and stained with 2% phosphotungistic acid and dried using infrared light. The particles were then observed under electron microscope.

### Drug loading study

The drug loading and quantification was performed by OPDA-derivatization method. For this purpose, known quantity of dried liposome was added to 100 µl of DMF and vortexed. To this, 100 µl of OPDA and 200 µl of PBS were added and the mixture was heated at 100 °C for 15 min. The samples were cooled in ice-bath and 1.6 ml of DMF was added to make it to 2 ml as a final volume. Earlier, a calibration curve was performed at different concentration of CDDP and analyzed through UV–Vis spectrophotometer (JASCO-V-730) at a wavelength of 706 nm.

### Cellular uptake of CD59sp-conjugated CDDP/miRNA-loaded liposomes

The cellular uptake study was first performed by confocal laser scanning microscopy (CLSM) method. Briefly, HeLa cells were seeded in 6-well plate with a cover-slip on it and the cells were incubated for 24 h at 37 °C. Next day, cells were treated with LP-miCDDP and CD/LP-miCDDP formulations, respectively for 2 h. The cells were washed 2 times and immediately incubated with Lysotracker Green^TM^ for 10 min. The cells were washed and fixed with 4% paraformaldehyde and stained with DAPI as a nuclear staining. The cover-slip containing cells were mounted on a glass slide and observed under microscope (Leica SP8).

The cellular uptake was further evaluated by flow cytometer. Briefly, HeLa cells were seeded in 6-well plate and the cells were incubated for 24 h at 37 °C. Next day, cells were treated with LP-miCDDP and CD/LP-miCDDP formulations, respectively for 2 h. The cells were washed and detached with trypsin/ethylenediaminetetraacetic acid mixture and centrifuged a 1200 rpm for 3 min. The pellet cells were reconstituted with the PBS and studied in BD FACSCalibur flow cytometer (BD FACS, NJ, USA).

### In vitro anticancer effect analysis

The in vitro anticancer effect of free miRNA and the formulations were evaluated by MTT assay. Briefly, briefly, 1 × 10^4^ HeLa cells were seeded in 96-well plate and the cells were incubated for 24 h at 37 °C. The cells were either treated with increasing concentration of lipofectamine-miRNA complex or free CDDP, miCDDP and CD/LP-miCDDP formulations or incubated for 24 h. The cells were washed two times with PBS and added with 20 µl of MTT solution that has a concentration of 5 mg/ml and incubated for 4 h at 37 °C. 100 µl of DMSO was added and incubated for 15 min and then absorbance was read at 570 nm using a microplate reader. The untreated cells were taken as a control and IC50 value were calculated from GraphPad Prism software.

### Apoptosis analysis–nuclear staining

The nuclear morphology of cancer cells after treatment was evaluated by Hoechst 33,342 staining. Briefly, 2 × 10^5^ HeLa cells were seeded in 96-well plate and the cells were incubated for 24 h at 37 °C. The cells were then treated with free CDDP, miCDDP and CD/LP-miCDDP formulations and incubated for 24 h. The cells were washed with PBS twice and fixed with 4% paraformaldehyde for 10 min. The cells were washed and then stained with 10 µg/ml of Hoechst 33342 solution for 15 min. The cells were washed three times and morphology was observed through IN Cell Analyzer 2000 (GE Healthcare Life Science, USA).

### Apoptosis analysis–flow cytometer

The quantitative apoptosis was performed by Annexin V-FITC and PI double staining followed by flow cytometer analysis. Briefly, 2 × 10^5^ HeLa cells were seeded in 96-well plate and the cells were incubated for 24 h at 37 °C. The cells were then treated with free CDDP, miCDDP and CD/LP-miCDDP formulations and incubated for 24 h. After 24 h, cells were scrapped gently and centrifuged at 1200 rpm for 3 min. The pellet cells were reconstituted with 100 µl of binding buffer and stained with 5 µl of Annexin V-FITC and PI and subjected to incubation for 15 min in the dark atmosphere. The volume was made up to 1 ml and studied using the in BD FACSCalibur flow cytometer (BD FACS, NJ, USA).

### Pharmacokinetic analysis

The pharmacokinetic analysis of free drug and drug-loaded formulations were studied in Sprague–Dawley rats (200 + 20 g). The rats were given free access to food and water until the study and caged in a standard atmosphere as per the guidelines set by the Institutional committee for animal care and handling. The rats were divided into 3 groups with 5 rats in each group. All the rats received the drug and formulation treatment via the tail vein injection. The CDDP is administered at a dose of 5 mg/kg while formulations were given 5 mg/kg equivalent dose. The blood samples were collected from the rats from retro-orbital plexus after 0.25 h, 0.5 h, 1 h, 2 h. 4 h, 6 h, 8 h, 12 h, 24 h, 36 h and 48 h, respectively. The heparin mixed samples were centrifuged at 10,000 rpm for 10 min and plasma was stored for further analysis in − 80 °C. 150 µl of plasma and 150 µl of ethanol was mixed and vortexed for 1 h. The mixture was centrifuged at 12,000 rpm for 15 min and the supernatant was used to calculate the CDDP concentration. The CDDP was measured by ion coupled plasma mass spectrometry (ICP-MS, Perkin-Elmer Corporation, USA).

### Statistical analysis

P value < 0.05 was considered statistically significant. Quantitative data were expressed as mean ± SD. Statistical comparisons were made by one-way ANOVA analysis and Student’s *t* test.

## Results

### Particle size and surface charge characterization of particles

**Fig. 2:**
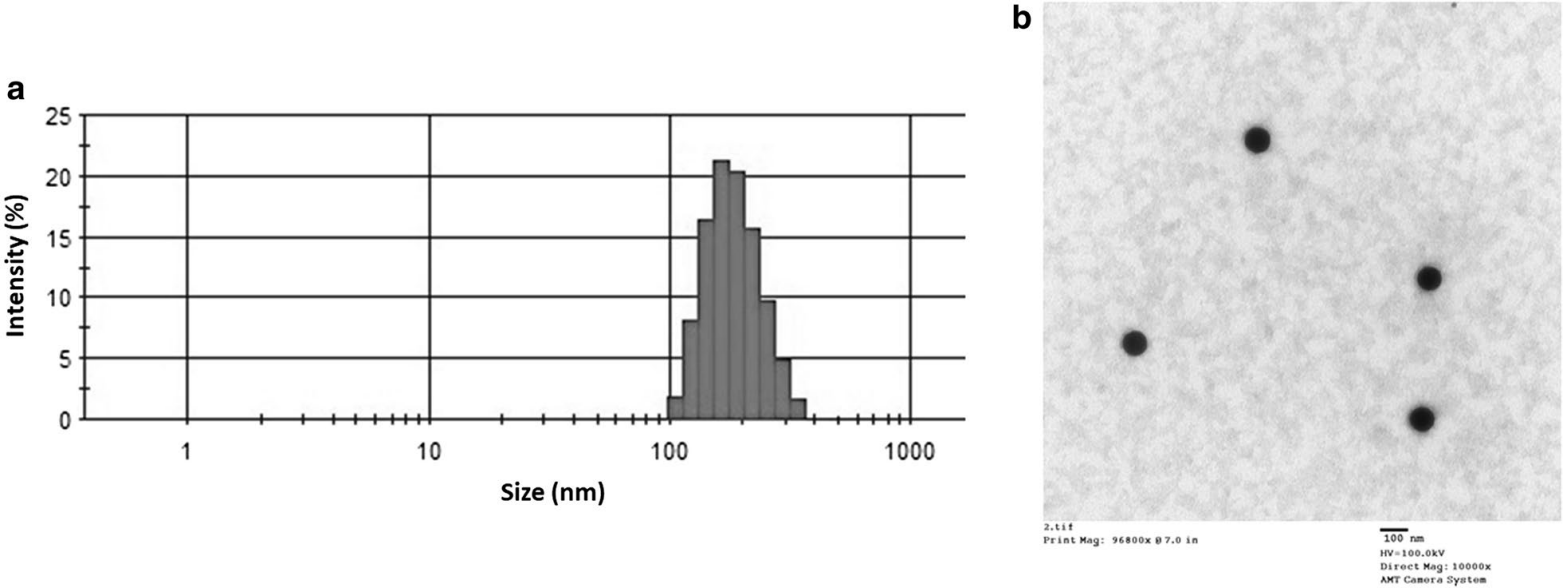
**a** Physicochemical characterization of CD/LP-miCDDP, particle size distribution using dynamic light scattering (DLS); **b** morphology analysis of CD/LP-miCDDP using transmission electron microscopy (TEM)

The man particle size and surface charge of particles were evaluated by DLS method. The mean particle size of LP-miCDDP was 145.2 ± 1.25 nm while the particle size increased to 168.4 ± 1.34 nm for CD/LP-miCDDP after the surface conjugation with CD59 antibody with a PDI of 0.216. CD/LP-miCDDP exhibited a surface charge of 16.2 ± 1.61 mV which could help to internalize the particles in the cancer cells (Fig. 2a). Several reports have provided evidences that the cellular uptake of nanoparticles is directly related to its size. The nanoparticles of size between 100 and 200 nm are reported to preferentially accumulate in the tumor tissues than the normal tissues owing to enhanced permeation and retention (EPR) effect. In this regard, CD/LP-miCDDP showed a smaller particle size with an optimal surface charge sufficient for enhanced accumulation in the tumor tissues. The surface morphology of the CD/LP-miCDDP was evaluated by TEM (Fig. 2b). As shown, particles were perfectly spherical in shape and well-dispersed on the copper grid. The surface modification of particle did not influence the shape of the particles.

### Stability and in vitro drug release study

**Fig. 3:**
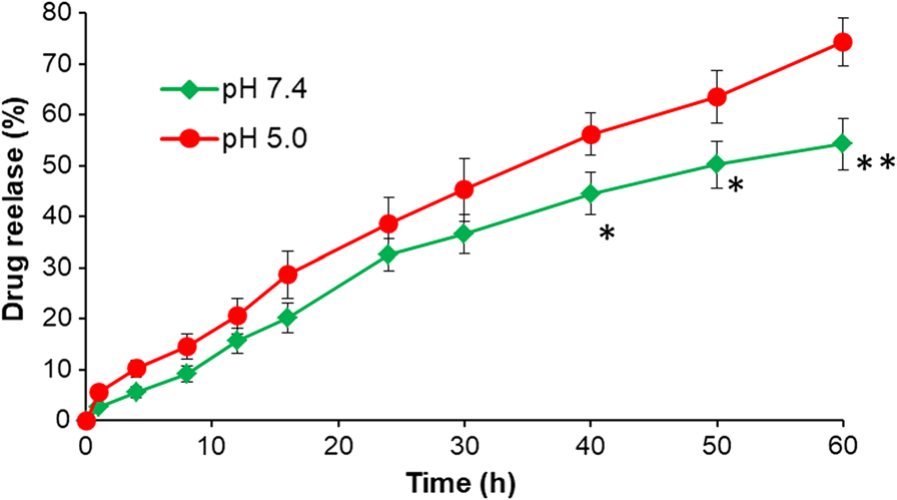
In vitro drug release study of CD/LP-miCDDP in pH 7.4 and pH 5.0 conditions. *p < 0.05, *p < 0.01 pH 7.4 vs pH 5.0

The stability analysis of CD/LP-miCDDP in PBS was evaluated using dynamic light scattering (DLS) analysis (Additional file 1: Figure S1). As shown, CD/LP-miCDDP showed excellent stability of particles at 4 °C for at least 10 days and the particle size of CD/LP-miCDDP did not change throughout the study period indicating no aggregation or degradation process. In vitro release of CDDP from CD/LP-miCDDP was performed by dialysis method (Fig. 3). The drug release study was performed in pH 7.4 and pH 5.0 conditions in order to simulate the physiological and acidic conditions. As shown, consistent release of CDDP was observed throughout the study period of 60 h in both the pH conditions. For most part of time, no significant difference in release of CDDP was observed from CD/LP-miCDDP between pH 7.4 and pH 5.0 while significant difference in release was observed between 40 and 60 h. A slightly higher drug release in pH 5.0 conditions might be attributed to the higher diffusion of drugs compared to that in pH 7.4 condition. Nonetheless, low drug release in physiological conditions might decrease the systemic adverse effect and allow its release in the acidic environment of tumor.

### CD/LP-miCDDP enhanced cellular accumulation in HeLa cells—CLSM and Flow cytometer analysis

**Fig. 4:**
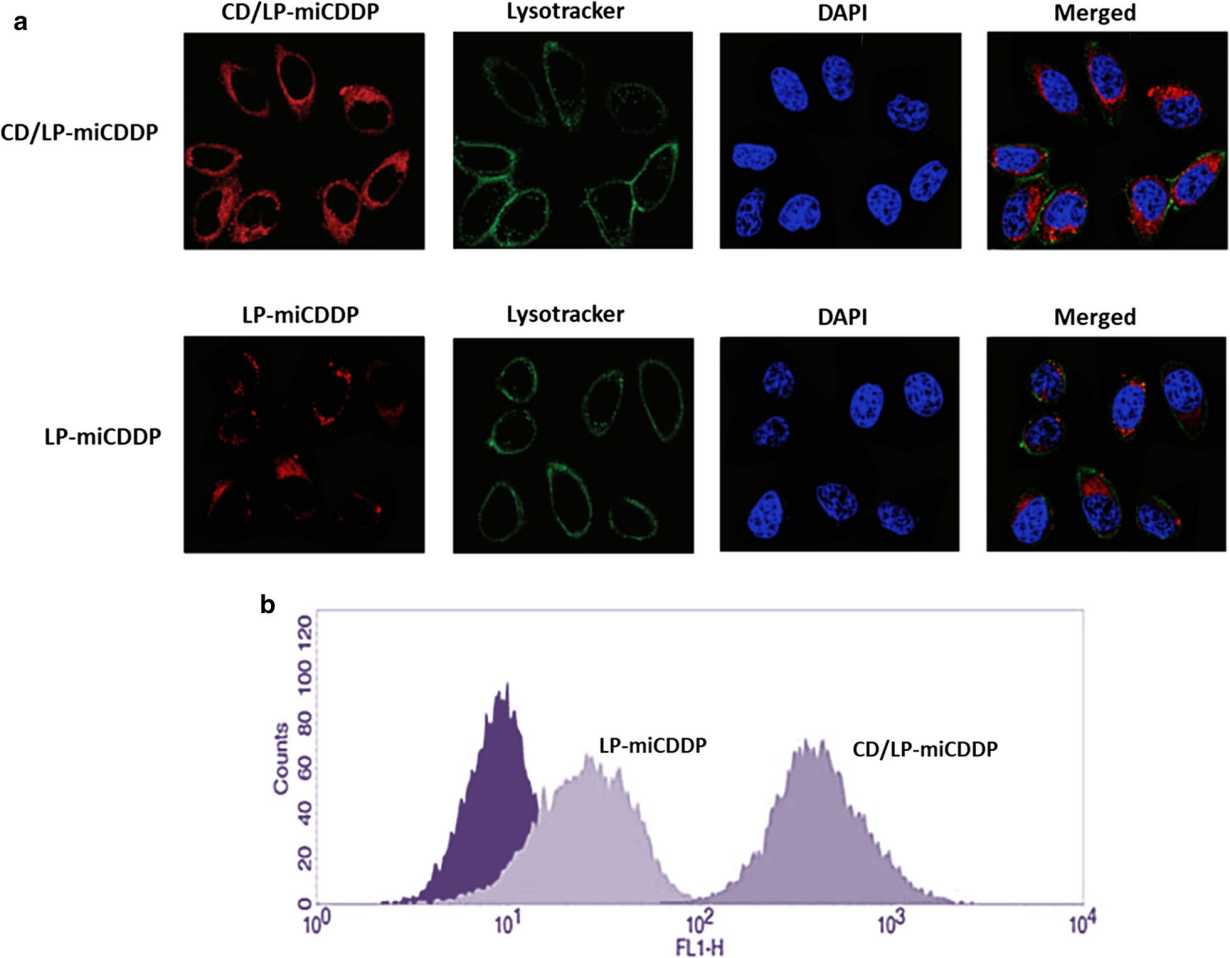
**a** Cellular uptake analysis of LP-miCDDP and CD/LP-miCDDP in HeLa cells using confocal laser scanning microscopy (CLSM). The Rhodamine B was used as a fluorescent tracker and the cells were stained with Lysotracker Green and DAPI as a respective lysosome and nucleus tracker; **b** flow cytometer analysis of cellular uptake after incubation of liposomes to cells for 2 h

The target specificity of CD59 antibody conjugated liposome was compared with the non-targeted liposome by means of confocal laser scanning microscopy (CLSM) (Fig. 4a). Rhodamine B was loaded in the liposome as a fluorescence tracker and lysosomes were stained with Lysotracker Green and nucleus was stained with DAPI (blue color). The HeLa cells were exposed with CD/LP-miCDDP and LP-miCDDP and incubated for 2 h. As shown, CD/LP-miCDDP exhibited a remarkably higher cellular accumulation in the cancer cells compared to that of LP-miCDDP treated cells. Merged image clearly indicates the presence of liposomes in the lysosome and cytoplasm region and none in the nucleus indicating a typical receptor-mediated cellular uptake. A substantially higher uptake in case of CD/LP-miCDDP was believed to be due to the CD59-based receptor uptake. As shown (Fig. 4b), CD/LP-miCDDP showed a significantly higher shift in the fluorescence histogram than that of non-targeted liposomes.

### MiR-1284 downregulates HMGB1 –Western blot analysis

**Fig. 5:**
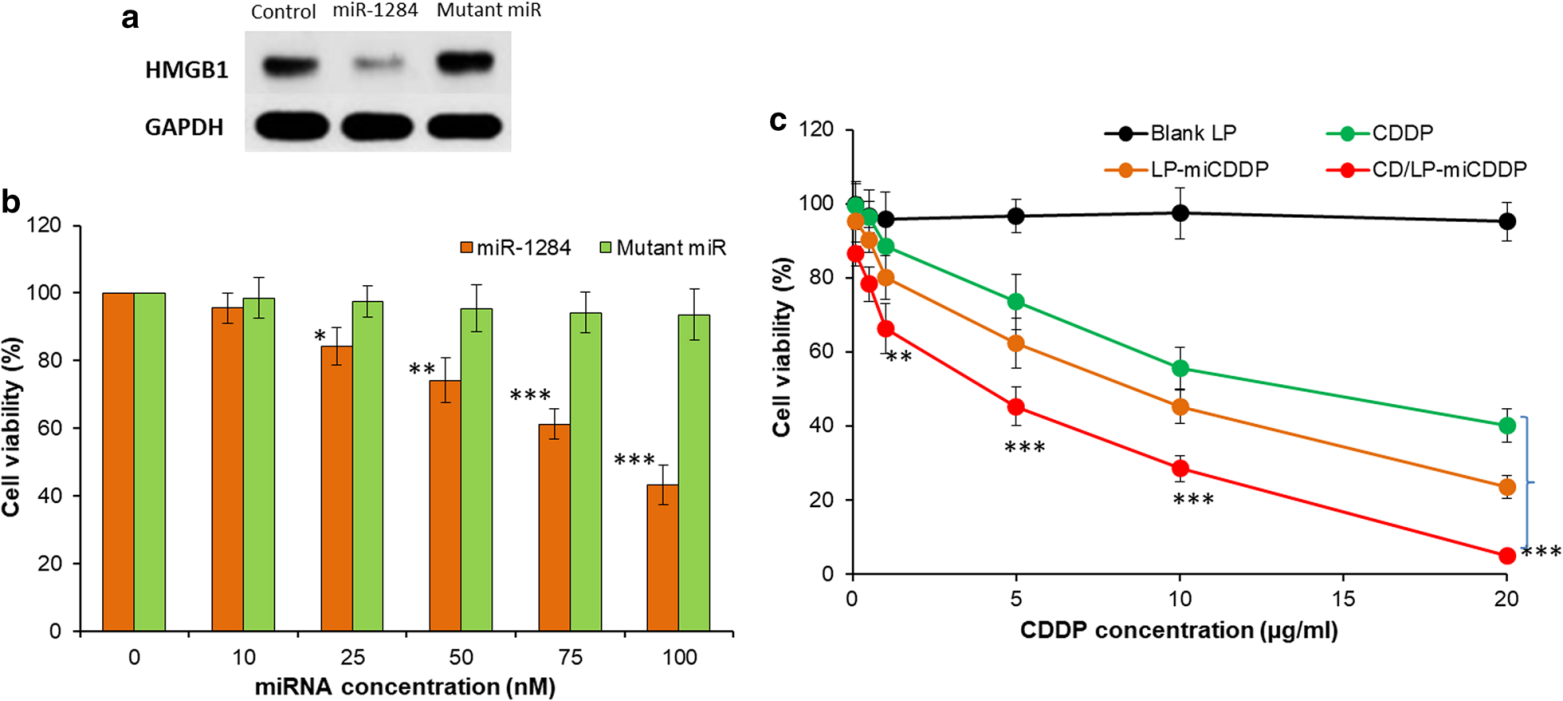
**a** cell viability analysis of miR-1284 and Mutant miR on the HeLa cancer cells in a dose-dependent manner; **b** cell viability analysis of free CDDP and formulations on the viability of cancer cells. The cell viability was determined by MTT assay. ***p < 0.0001, CD/LP-miCDDP and vs free CDDP

Keeping this in mind, miR-1284 effect on the cervical cancer cells were analyzed by western blotting (Fig. 5a). As expected, protein levels of HMGB1 were significantly downregulated in the HeLa cells by miR-1284 mimics while mutant miR did not have any effect on the protein indicating the potential of miR-1284.

### Combination of miR-1284 and CDDP in the cell viability

Here, we have performed a cell viability analysis of effect of miR-1284 mimics a mutant miR on the HeLa cancer cells. As shown (Fig. 5b), miR-1284 showed a typical concentration-dependent cell killing effect in the cervical cancer cells whereas mutant miR did not have any effect on the proliferation of cancer cells. Next, we have evaluated the combinational effect of miR-1284 and CDDP in HeLa cancer cells. We have treated the cells were free CDDP, LP-miCDDP and CD/LP-miCDDP and incubated for 24 h (Fig. 5c). As shown, all the formulation exhibited a concentration-dependent cytotoxic effect in the proliferation of cervical cancer cells.

### Apoptosis analysis–Hoechst 33342 staining

**Fig. 6:**
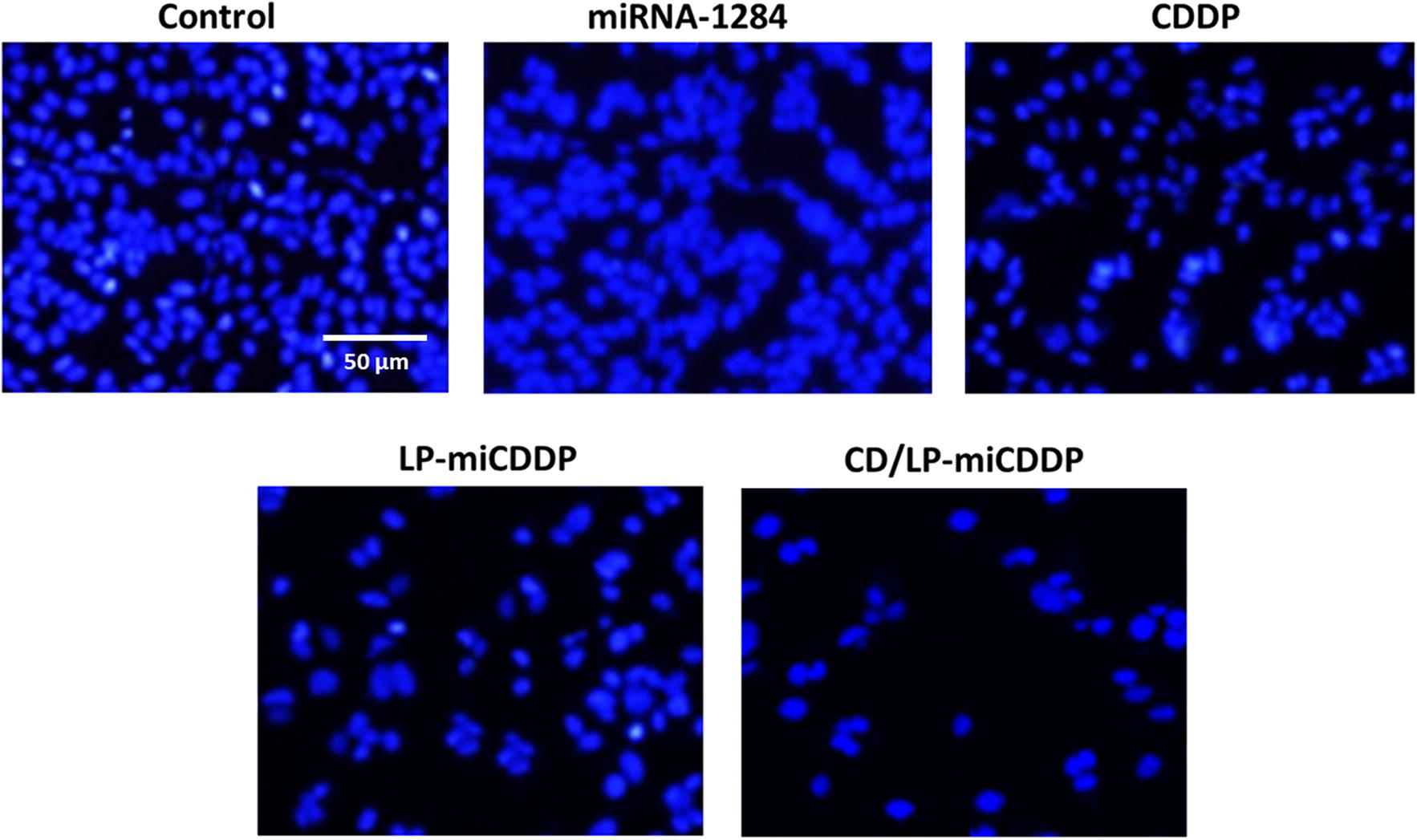
Apoptosis analysis of HeLa cancer cells after treatment with miR-1284, CDDP, LP-miCDDP and CD/LP-miCDDP using Hoechst 33342 staining

The apoptotic effect of each formulation was first determined by Hoechst 33342 staining analysis (Fig. 6). The cells were treated with respective formulations and then stained with Hoechst 33342 and observed for changes in cell morphology in the fluorescence microscope. As shown, cell nuclei were perfectly round and lots of cells were attached firmly with the culture plate. However, cells treated with miR-1284 and CDDP resulted in slight apoptosis as observed from the distorted cells and cells undergoing apoptosis. Most remarkable effect was seen in cells treated with CD/LP-miCDDP where remarkable cell/nuclei rupturing and apoptotic bodies were observed. The morphological changes in the cell nuclei of CD/LP-miCDDP treated cells were markedly greater than control cells. Moreover, remarkable decrease in the total cell attached to the plate was observed in CD/LP-miCDDP group indicating the appreciable apoptosis effect of the formulation in the cervical cancer cells.

### Apoptosis analysis—flow cytometer

**Fig. 7:**
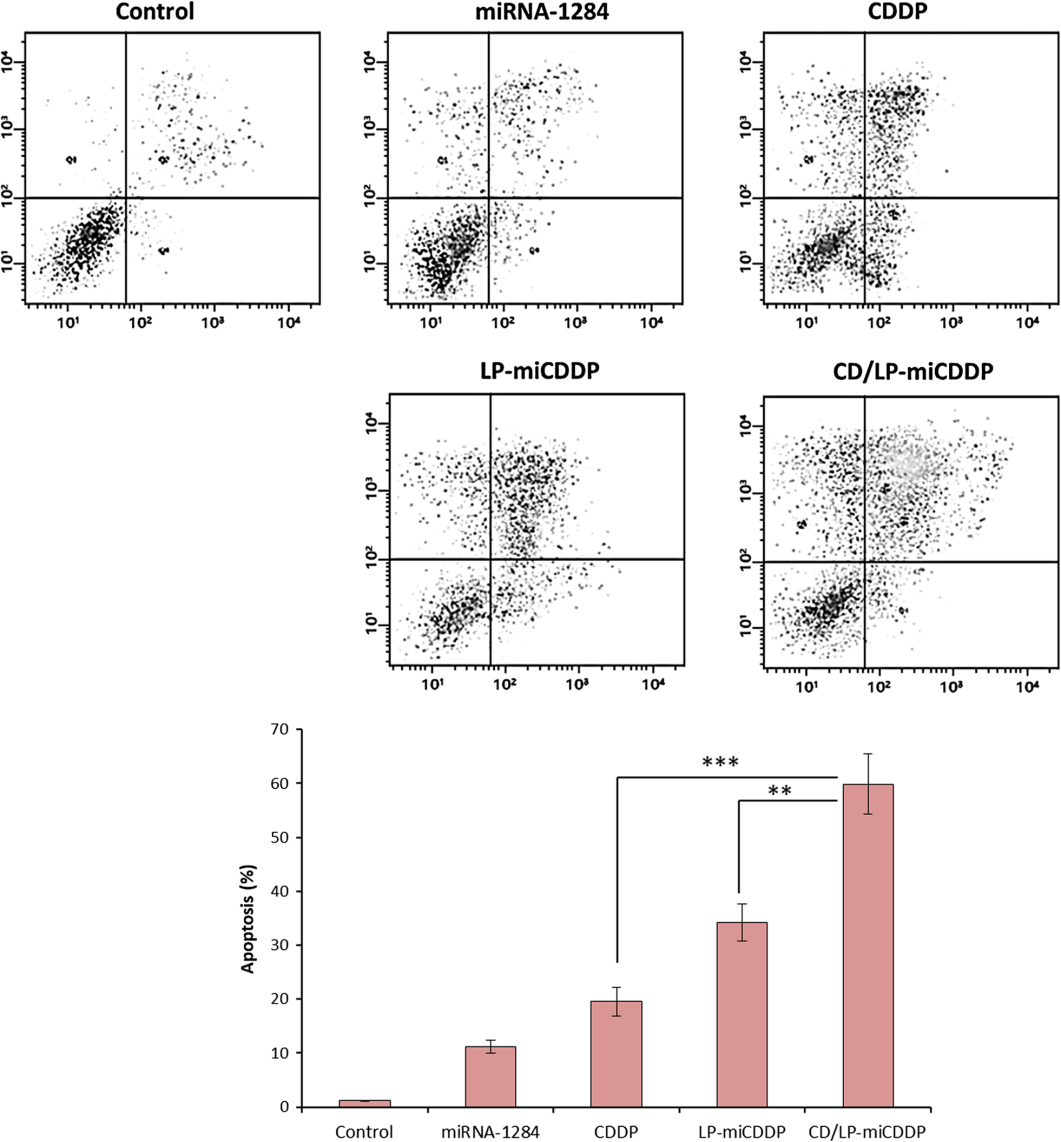
Quantitative apoptosis analysis of of HeLa cancer cells after treatment with miR-1284, CDDP, LP-miCDDP and CD/LP-miCDDP. The cells were stained with Annexin V-FITC and PI and 10,000 cell events were observed at flow cytometer. ***p < 0.0001, CD/LP-miCDDP vs free CDDP; **p < 0.001, CD/LP-miCDDP and vs LP-miCDDP

The quantitative apoptosis effects of formulations were studied by flow cytometer after staining with Annexin V-FITC and PI (Fig. 7). The Annexin V-FITC detects the early apoptosis cells by binding with high affinity to the extracellular phosphatidylserine which is exposed in the cells undergoing apoptosis, while, PI penetrates the dead cells through damaged membrane and exhibited red fluorescence. As shown, CD/LP-miCDDP exhibited the maximum apoptosis effect (~ 60%) compared to CDDP (~ 20%) or miR-1284 (~ 12%) treated cells indicating the superior anticancer effect in the cancer cells. LP-miCDDP also exhibited the appreciable apoptosis effect of ~ 35% indicating the potential of combinational therapeutics and carrier-based drug delivery.

### Pharmacokinetic analysis in animals

**Fig. 8:**
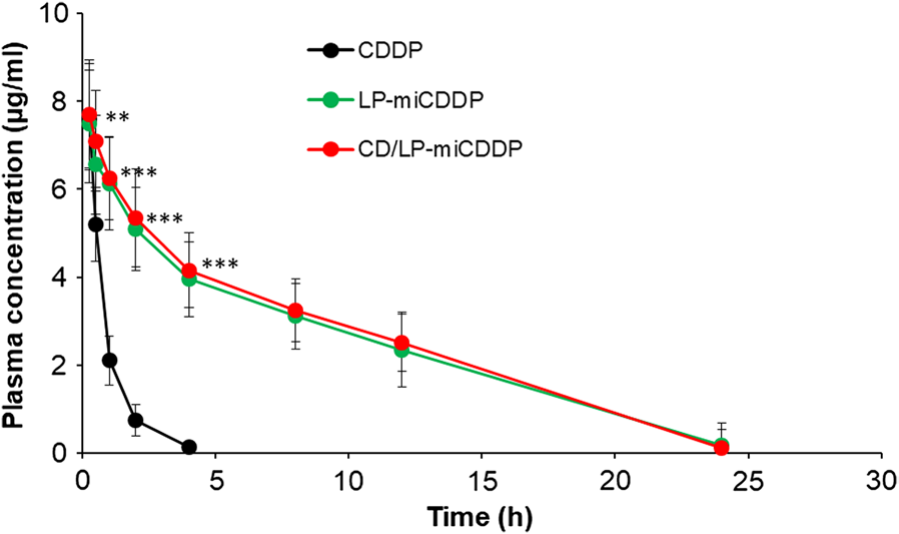
Plasma concentration–time profile of free CDDP, LP-miCDDP and CD/LP-miCDDP. The free drug and formulations were administered in SD rats by tail-vein injection and studied until 24 h. ***p < 0.0001, CD/LP-miCDDP and vs free CDDP

Following the enhanced anticancer effect of formulations, we have evaluated the pharmacokinetic performance of free drug and formulations in SD rats (Fig. 8). Animals were divided into 3 groups and formulations were administered. As shown, free CDDP was immediately removed from the blood circulation within 4 h of administration consistent with many reports regarding the free drug behavior. On the other hand, LP-miCDDP and CD/LP-miCDDP significantly prolonged the blood circulation of encapsulated drug until at least 24 h after the administration.

## Discussion

Cervical cancer is one of the gynecological cancer responsible most cancer-related deaths in women. Though radiotherapy and surgery are common option in case of advanced cervical cancer, however this standard treatment is unavailable in many developing countries. Based on the guidelines of NCCN for cervical cancer, cisplatin (CDDP) is considered as a first line of treatment option. However, chemotherapy with CDDP results in severe dose-limiting toxicity such as nephrotoxicity or hepatotoxicity and leads to severe drug resistance. Therefore, potential therapeutic approaches by which drug toxicity could be reduced and chemosensitivity of CDDP towards cervical cancer could be increase is perused continuously. In this study, we have selected miR-1284 to increase the chemosensitivity of CDDP for the treatment of cervical cancers. We have prepared unique CD59sp-conjugated CDDP/miR-1284-loaded liposomes for the effective treatment of cervical cancers.

A substantially higher uptake in case of CD/LP-miCDDP was believed to be due to the CD59-based receptor uptake. The CD59 antibody will recognize the corresponding receptor overexpressed in the cancer cells that promote the enhanced internalization of CD/LP-miCDDP in the cancer cells (Yang et al. 2017). The quantitative analysis of cellular uptake was performed by flow cytometer. A significant shift in the fluorescence histogram is proof of its enhanced cellular accumulations in the HeLa cells. These results were consistent with the previous reports of higher cellular uptake and higher therapeutic efficacy with CD59-tagreted carrier system in the cancer cells (Wang et al. 2017).

Towards the treatment of any cancer, chemotherapy is effective in the beginning however it loses its effectiveness due to the development of chemoresistance over a period of time. CDDP though very potent develops chemoresistance and therefore required to give higher doses which in turn results in severe toxicity. In this regard, it has been reported that miR-1284 could modulate the drug resistance in gastric cancer cells by targeting EIF4 A1 (Huang et al. 2017; Wei et al. 2019). As expected, protein levels of HMGB1 were significantly downregulated in the HeLa cells by miR-1284 mimics while mutant miR did not have any effect on the protein indicating the potential of miR-1284. It is worth noting that HNGB1 has been demonstrated to possess oncogenic role in human cancers (Cao et al. 2016).

Earlier reports have revealed that downregulation of HMGB1 could lead to the inhibition of cell proliferation. Results clearly highlight our expectation that downregulation of HMGB1 could be directly related to the proliferation of cervical cancer cells (Wu and Yang 2018; Lv and Guan 2018). It is worth noting that CD59-antibody conjugated formulation exhibited a significantly lower cell viability compared to that of free CDDP. The combination of miR-1284 and CDDP could result in a synergistic anticancer effect on the cervical cancer cells. We expect that miR-1284 might increase the chemosensitivity of HeLa cancer cells and thereby leading to enhanced cell killing effect. It must be noted that the CD/LP-miCDDP exhibited significantly lower cell viability than that of LP-miCDDP attributed to its higher cellular internalized as observed in CLSM and flow cytometer analysis. The IC50 value of CDDP, LP-miCDDP and CD/LP-miCDDP was observed to be 12.4 µg/ml, 7.23 µg/ml and 3.12 µg/ml, respectively, consistent with the cell viability analysis. Blank liposome did not evoke any cytotoxic effect in all tested concentration indicating the biocompatible and safe properties of carrier system. The enhanced anticancer effect of CD/LP-miCDDP was mainly attributed to increased chemosensitivity of CDDP towards the HeLa cells sue to the presence of miR-1284 that acted in a synergistic manner (Pan et al. 2016). Besides, CD59 was specific towards its receptor overexpressed in the cervical cancer cells that resulted in enhanced accumulation of liposomes and exhibited higher therapeutic effect.

LP-miCDDP and CD/LP-miCDDP significantly prolonged the blood circulation of encapsulated drug until at least 24 h after the administration. The AUC_(o-t)_ of CD/LP-miCDDP showed a 6.9 fold higher value than that of free CDDP. Similarly, CD/LP-miCDDP showed an eightfold decrease in the clearance (CL) and 3.6-fold higher t_1/2_ compared to that of free CDDP. A higher plasma concentration will eventually benefit the anticancer therapy as there will be more chances of tumor accumulation. At the same time, it will minimize the chance of off target to other organs that will substantially reduce the severe adverse effects associated with the CDDP.

In summary, CD/LP-miCDDP was effective carrier for the antitumor co-delivery of miRNA-1284 and CDDP against cervical cancers. CD/LP-miCDDP was successfully internalized by HeLa cells. Co-delivery of MiR-1284 and CDDP showed a synergistic effect in suppressing the cervical cancer cells by the downregulation of HMGB1. The synergistic anticancer efficacy of CD/LP-miCDDP was further demonstrated by maximum apoptotic cells (flow cytometer and Hoechst staining). CD/LP-miCDDP exhibited a significantly prolonged blood circulation potential compared to that of free CDDP. Overall, present study provides a promising platform for the co-delivery of therapeutic gene and small molecule in the treatment of cervical cancer, and possibly other types of cancer as well. Futuristic studies will be focused on testing on clinically relevant animal model and studying the benefits in other cancers.

## Supplementary information

**Additional file 1: Figure S1.** Stability analysis of CD/LP-miCDDP in PBS buffer system.

## Data Availability

Not applicable.

## Abbreviations

CDDP: Cisplatin
LP: Liposome
miR: miRNA
CD/LP-miCDDP: CD59 antibody-conjugated miRNA-1284/CDDP-loaded liposomes
LP-miCDDP: miRNA-1284/CDDP-loaded liposomes
EPR: Enhanced permeation and retention
