# Supplementary material for: CD59 receptor targeted delivery of miRNA-1284 and cisplatin-loaded liposomes for effective therapeutic efficacy against cervical cancer cells
Source: AMB Express. 2020 Mar 17;10:54. doi: 10.1186/s13568-020-00990-z (PMC7078418; doi:10.1186/s13568-020-00990-z)
Supplement: Supplementary file 1 — Additional file 1: Figure S1. Stability analysis of CD/LP-miCDDP in PBS buffer system. [file 13568_2020_990_MOESM1_ESM.docx]

**CD59 receptor targeted delivery of miRNA-1284 and Cisplatin-loaded liposomes for effective therapeutic efficacy against cervical cancer cells**

Li Wang^1^, Ting-Ting Liang^2^*

^1^Department of Pharmacy, Jining No.1 People's Hospital, Jining 272011, Shandong, China

^2^Department of Obstetrics and Gynecology, Weifang No.2 People’s Hospital, Weifang 261041, Shandong, China

***Corresponding author:**

Ting-Ting Liang, MD

Department of Obstetrics and Gynecology,

Weifang No.2 People’s Hospital, No. 7 Yuanxiao Street,

Kuiwen District, Weifang 261041, Shandong, China

Tel/Fax: 0086-0536-8233405

Email: [TrevonBraunrba@yahoo.com](mailto:TrevonBraunrba@yahoo.com)


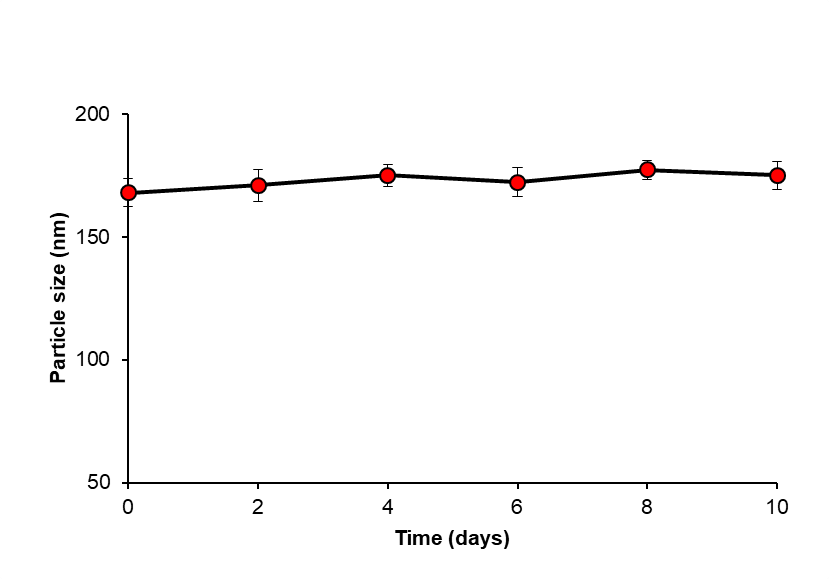


Figure S1: Stability analysis of CD/LP-miCDDP in PBS buffer system
